# Offense and defense: itaconate mediates bidirectional immune regulation of host-bacteria interaction

**DOI:** 10.1186/s12929-026-01266-7

**Published:** 2026-06-26

**Authors:** Zhaoyue Men, Zishen Lin, Zhe Wang, Peng Tan, Shuyang Yu, Xi Ma

**Affiliations:** 1 State Key Laboratory of Animal Nutrition and Feeding, College of Animal Science and Technology, China Agricultural University, Beijing, 100193 China; 2 College of Animal Sciences, Fujian Agriculture and Forestry University, Fuzhou, Fujian 350002 China; 3 State Key Laboratory of Animal Biotech Breeding, College of Biological Sciences, China Agricultural University, Beijing, 100193 China

**Keywords:** Itaconate, Pathogenic bacteria, Host-bacteria interaction, Bacterial disease, Immunity, Immune modulation

## Abstract

Itaconate has garnered significant attention in recent years due to its immunomodulatory and antimicrobial functions. During inflammation and pathogenic infections, itaconate is formed through the decarboxylation of *cis*-aconitate in the mitochondrial tricarboxylic acid cycle and accumulates in large quantities to counteract excessive inflammation and pathogenic infections. However, many pathogenic bacteria have also evolved pathways to directly degrade itaconate or indirectly resist its stimulatory effects. We first review the research history and metabolic pathways of itaconate. Then we focus on exploring its direct mechanism of inhibiting pathogenic bacteria growth and reproduction by post-translational modification of metabolic enzymes such as isocitrate lyase, aldolase, and IMP dehydrogenase. Additionally, we examine its indirect mechanism of coordinating immune cell functions to eliminate pathogenic bacteria. Pathogenic bacteria counteract this by directly degrading itaconate through the IcT-IcH-CcL cascade reaction or by adapting through metabolic reprogramming to enable chronic infection. Subsequently, we discuss the spatiotemporal specificity of itaconate during the early and late stages of pathogenic bacteria infection, highlighting its role in regulating immune defense strategies at different phases. Finally, we discuss the potential and limitations of itaconate-related interventions as adjunctive strategies for bacterial disease control, particularly in the context of drug-resistant infections. This review elucidates the mechanism of itaconate in host-microbial crosstalk from the perspective of bidirectional resistance between host and bacteria, emphasizing its crucial role as a metabolic messenger in mediating co-evolutionary, co-developmental, and co-metabolic interactions between the host and bacteria. Current evidence of itaconate-mediated bidirectional interactions may help guide future mechanistic studies and the development of itaconate-related adjunctive strategies for bacterial disease control. Further *in vivo*, clinical, and field validation is still required before these findings can be translated into therapeutic or agricultural applications.

## Introduction

Hosts and microbes secrete various types of metabolites which are considered to be a kind of signal molecules between them. Such mutual signals may lead to synergistic or antagonistic results, thus greatly influencing physiological processes in the host organism and its pathogenesis [1, 2]. Itaconate (ITA), is one of the most important immunomodulatory molecules involved in host-microbial communication. In mammals it is derived by myeloid cells via alterations of their tricarboxylic acid (TCA) cycle metabolism and its production increases after lipopolysaccharide (LPS) treatment, the aconitate decarboxylase 1 (ACOD1, also known as immune response gene 1 (IRG1)) catalyses the transformation of *cis*-aconitate to high amounts of ITA [3]. ITA can also be the microbial metabolite. Fungi are identified to be a generator of ITA in the naturally or artificially genetic editing ways, such as *Aspergillus terreus* (*A. terreus*), filamentous fungus *Trichoderma reesei*, and yeast species [4]. Recently, ITA is regarded as a metabolic messenger, playing important roles in antibacterial and reconstruction of microbiome, antiviral, and response to immune and inflammation [5–8].

Host-bacteria interaction directly influence immune homeostasis [9–12]. On one hand, microbiota and their metabolites modulate the development, differentiation, and function of host immune cells, inducing metabolic and epigenetic reprogramming, as well as regulating inflammatory responses and signal transduction [13, 14]. On the other hand, the host also secretes various antimicrobial molecules as “metabolic messengers” to regulate pathogenic bacteria. In addition to ITA, host cells produce defensins, cathelicidins, and secretory immunoglobulin A (sIgA), which collectively exert antibacterial effects [15, 16]. ITA possesses both direct bactericidal and bacteriostatic effects against pathogenic bacteria and can interfere with bacterial metabolism through post-translational modifications or competitive binding mechanisms [17, 18].

However, the role of ITA in pathogenic bacterial infections is complex. For example, in *Staphylococcus aureus* (*S. aureus*) infection, ITA restricted bacterial growth and virulence yet facilitated long-term pathogen-host coexistence, resulting in chronic infection [19, 20]. This duality-ITA’s antimicrobial activity on one hand and its utilization by certain pathogens as a carbon source on the other-reflects the intricate co-evolutionary, co-metabolic, and mutually regulatory relationship between host and bacteria.

To better understand the role of ITA as a signaling molecule regulating host-bacteria interactions, we summarize the research history and metabolic pathways of ITA, outline its direct and indirect antibacterial mechanisms, and describe the strategies by which pathogenic bacteria utilize ITA or tolerate its stimulation. We also examine the spatiotemporal specificity of ITA’s effects during different stages of infection and discuss current applications and limitations. Through a bidirectional perspective, this review highlights the critical role of itaconate in mediating host-bacteria interaction, revealing the complex interplay of co-metabolism, co-development, and co-evolution in the ongoing host-bacteria arms race. Together, these studies and perspectives may provide useful directions for future investigations into biological mechanisms related to ITA and its potential relevance to clinical, veterinary, and livestock settings.

## Research history and metabolic pathways of ITA

### Research history of ITA

**Fig. 1:**
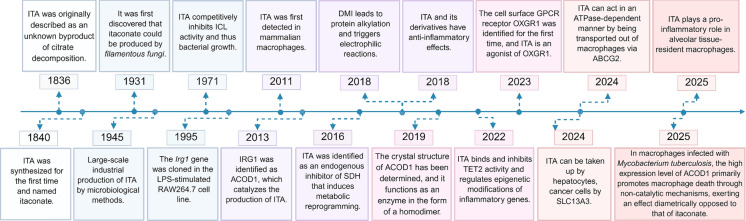
The research history of ITA. ITA was initially discovered as a chemical derivative and later found to be biosynthesized by microbes. Its identity shifted significantly in the twenty-first century with the recognition of ITA as an endogenous immunometabolite produced by macrophages via the IRG1/ACOD1 enzyme. Subsequent research uncovered its immunoregulatory functions, including SDH inhibition, activation of NRF2 and ATF3 pathways, and signaling through the surface receptor OXGR1 to drive mucociliary defense. Subsequently, the crystal structure of ACOD1 was determined, deepening our understanding of the formation of endogenous ITA. Recent studies identified ABCG2-mediated transport of ITA, establishing its role as a paracrine signal. Emerging evidence has highlighted the context-dependent pro-inflammatory role of ITA and the non-enzymatic role of ACOD1 in inducing macrophage cell death, thus highlighting the dual role of the protein in immune responses. Schematics were created with BioRender.com

ITA, also known as methylenesuccinic acid, possesses a trifunctional structure featuring an unsaturated double bond and two carboxyl groups. In recent years, growing evidence has revealed its profound biological functions and therapeutic potential in humans and animals, beyond its traditional role as a chemical synthesis intermediate in industry. Here, we provide a comprehensive overview of the major milestones in the research history of ITA (Fig. 1), illustrating the progressive elucidation of its bidirectional “offense and defense” functions in host-bacteria interaction.

#### Discovery and isolation of ITA (nineteenth-twentieth century)

ITA was first discovered in the nineteenth century, isolated and named after the thermal decomposition of citric acid [4]. The synthesis and industrial production of ITA commenced in the twentieth century. In 1931, Kinoshita et al. identified a filamentous fungus, *Aspergillus itaconicus*, capable of producing ITA, marking the first report on the natural biosynthesis of ITA from microorganisms [16]. Subsequently, various microbial strains, including *A. itaconicus*, *Escherichia coli* (*E. coli*), *Saccharomyces cerevisiae*, and *Aspergillus niger*, were found to produce ITA through natural or engineered biosynthesis, paving the way for its broad industrial production and application [21].

#### Functional and mechanistic exploration of ITA (early twenty-first century–2018)

By the twenty-first century, several research groups, such as the team led by Cheryl L. Strelko, began to recognize ITA as an important endogenous metabolite in mammals. Specifically, ITA was found to be produced in large quantities by macrophages upon extracellular stimulation [22].

This response is considered part of the immune reaction, establishing ITA as a representative immunomodulatory molecule [4]. *Irg1* was discovered as early as 1995 in the RAW 264.7 cell line stimulated by LPS, although the function of its encoded protein remained unknown at the time [4]. Subsequent work by Michelucci and colleagues demonstrated that IRG1 encodes the enzyme aconitate decarboxylase 1 (ACOD1), which catalyzes the decarboxylation of*cis*-aconitate to ITA in mammals [3]. After 2016, the functions and mechanisms of ITA were further elucidated. For example, Lampropoulou et al. showed that endogenous ITA inhibits succinate dehydrogenase (SDH)-mediated succinate oxidation, thereby regulating macrophage metabolism, mitochondrial respiration, and inflammatory cytokine production [23]. By 2018, it was discovered that the ITA derivative 4-OI can alkylate KEAP1, thereby activating the NRF2 signaling pathway [24]. DMI can react with and deplete intracellular glutathione, a process that induces electrophilic stress, leading to the downregulation of IκBζ and upregulation of ATF3 [25]. These findings led to the recognition that ITA and its derivatives possess anti-inflammatory effects.

#### In-depth research on ITA (2019–present)

Since 2019, research on ITA has entered a phase of rapid advancement. The crystal structures of human and mouse ACOD1 were resolved, revealing the structural basis of its catalytic activity [26]. Concurrently, the application of advanced proteomic technologies enabled the identification of multiple ITA-interacting proteins, greatly expanding the understanding of ITA’s regulatory network [27, 28]. Subsequent studies demonstrated that ITA is not confined to macrophages but can be taken up and transported to various cell types via specific transporters, acting as a paracrine signaling molecule to mediate intercellular communication [29–33]. A breakthrough in 2023 revealed that ITA directly activates a key host defense mechanism-mucociliary clearance-through binding to a cell-surface receptor, opening a new window into the role of ITA in innate immunity [34]. However, further studies have shown that ITA’s effects are not uniformly anti-inflammatory or protective: in certain tissue microenvironments, such as alveolar-resident macrophages, ITA can exhibit pro-inflammatory properties [35]; in the context of specific pathogenic bacteria infection, ACOD1 can also influence host cell survival through non-catalytic pathways [36]. Collectively, these findings indicate that ITA acts as a bidirectional modulator in host-bacteria interactions, encompassing both defense and regulation, as well as protective and detrimental effects.

### Metabolism of ITA in animals

The bidirectional immunomodulatory functions of ITA in host-bacteria interaction are intimately linked to its anabolic and catabolic metabolism in animals. Understanding these metabolic pathways helps reveal the spatiotemporal rules governing ITA activation and clearance under different physiological and pathological conditions.

#### Regulation of endogenous ITA production

ITA is synthesized by the mitochondrial enzyme ACOD1, also known as IRG1 or CAD, which catalyzes the decarboxylation of cis-aconitate in a branch of TCA cycle [3]. ACOD1 is a homodimer with its active site located at the junction of two distinct subdomains [26]. The sequence and structure of ACOD1 are highly conserved across evolutionary lineages, from fungi to zebrafish to humans; however, the catalytic activity of murine Acod1 is higher than that of human ACOD1, likely due to amino acid differences near the active site that affect active-site opening and closing efficiency and thereby influence endogenous ITA production [26, 37–39]. The Asn152Ser variant leads to increased ACOD1 activity and is particularly common in African populations [26]. Human ACOD1 is unique in containing methionine instead of isoleucine at residue 154, which results in lower enzymatic activity compared to that in mouse and other species [37]. Differences in the crystal structure and cross‑species sequence specificity of the ACOD1 protein reveal potential variations in its enzymatic activity, which are highly relevant to ITA production and immunomodulatory diseases.

**Fig. 2:**
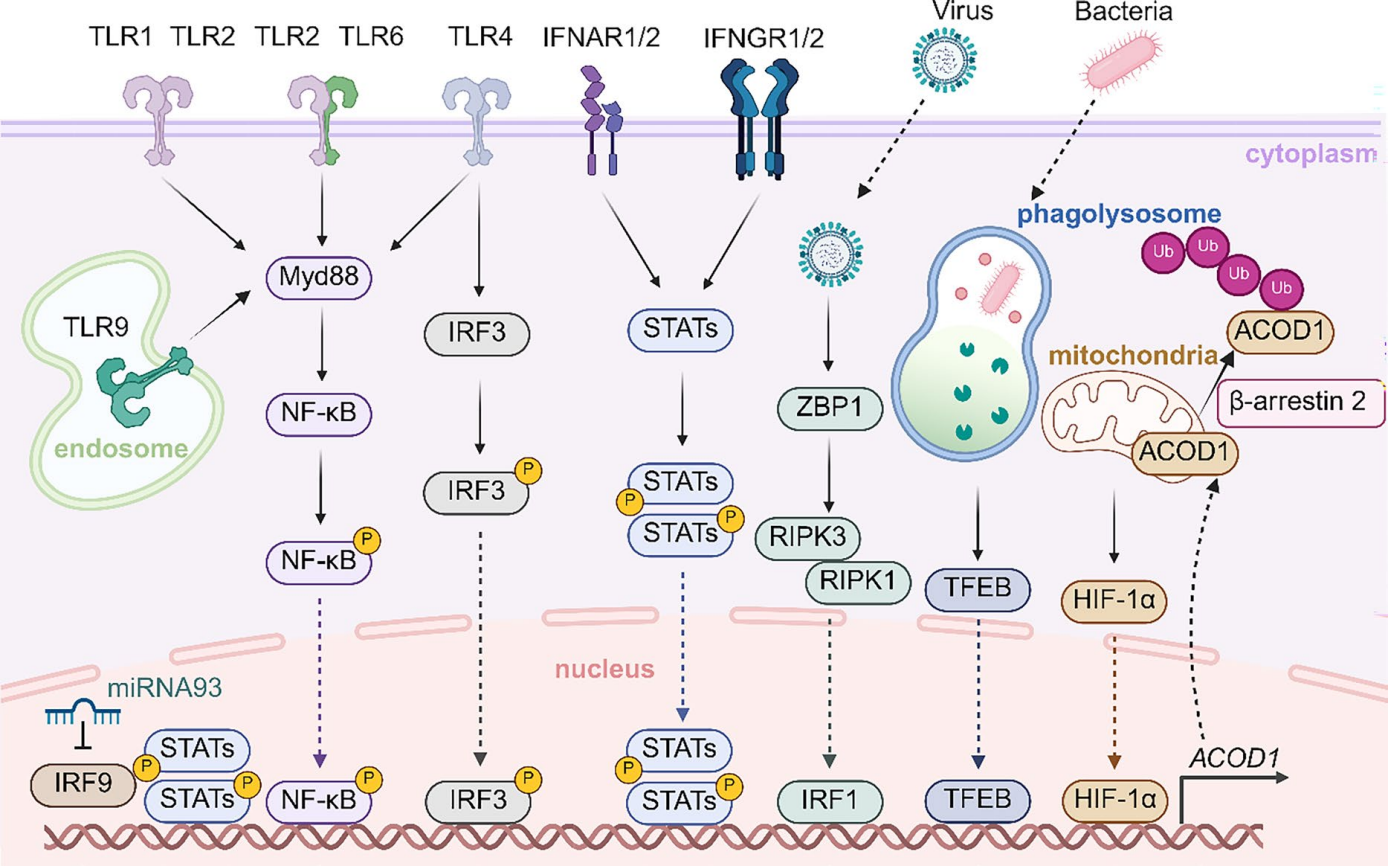
Mechanisms on membrane proteins and transcription factors governing ACOD1 expression. Synthesis of ITA is mainly regulated at the transcriptional level by induction of its biosynthetic enzyme ACOD1. This figure shows the primary pathways upstream of ACOD1 activation. Pathogen extracellular stimuli (e.g., viruses, bacteria) are detected via various membrane and endosomal receptors, including Toll-like receptors (TLRs, such as TLR1/2, TLR3, and TLR4) and interferon receptors (e.g., IFNAR1/2, IFNGR1/2). These signals converge on major transcriptional regulators like NF-κB, IRF3, and STATs, which then translocate into the nucleus to drive transcription of the *ACOD1* gene. The pathways highlighted here integrate diverse immune signals to precisely control the production of the immunometabolite ITA. Schematics were created with BioRender.com

As many studies indicated, there are several factors influencing ITA development. Firstly, is a flux of carbons through the TCA cycle, which indirectly regulates ITA’s *de novo* synthesis. In hypoxia, reductive carboxylation by the enzyme isocitrate dehydrogenase (IDH) increases in macrophages, increasing the concentration of *cis*-aconitate, thereby promoting ITA production. Interestingly, ITA itself inhibits IDH2-mediated reductive carboxylation, creating a feedback loop that limits its own synthesis and reduces levels of other key immune-related metabolites, such as citrate and 2-hydroxyglutarate [40]. This indicates that although ACOD1 catalyzes the direct decarboxylation of *cis*-aconitate to produce ITA, the carbon flux through the TCA cycle strongly influences this biosynthetic route. It is also worth noting that in the healthy state, macrophages show little to no production of ACOD1 or ITA [3, 41]. However, upon challenge with an inflammatory stimulus or pathogen (e.g., LPS and other TLR agonists such as Pam3Cys-Ser-(Lys)4 or lipoteichoic acid), *ACOD1* transcription is rapidly upregulated [4]. The rapid induction leads to an accumulation of ITA at millimolar concentrations within a few hours, as reported by authors [4]. A number of transcription factors are known to regulate the expression of *ACOD1* [4], such as JUN, IRF1, IRF3, IRF9, STAT3, C/EBPβ, TFEB, HIF-1α, and NF-κB. Yet little is known about the post-translational regulation of ACOD1 activity. As an adaptor protein, arrestin beta 2 (β-arrestin 2) promotes the ubiquitination of ACOD1, thereby leading to a reduction in ITA in macrophages [42]. Taken together these findings show that there are multiple pathways involved in controlling ACOD1. We summarize the mechanisms regulating ACOD1 at the transcriptional and protein levels in Fig. 2.

However, it is important to note that the expression level of ACOD1 cannot be entirely equated with that of ITA, as ACOD1 possesses non-enzymatic functions. During *M. tuberculosis* infection, ACOD1 caused HSP70 degradation without requiring its enzymatic activity and made the lysosomal membrane more permeable, leading to macrophage death [36]. In contrast, the ITA derivative 4-OI increased the survival of infected cells [36]. Given that ACOD1 exhibits non-enzymatic functions, we should exercise caution when interpreting differences in the expression or function of ACOD1 and ITA.

#### Catabolism of ITA in animals

**Fig. 3:**
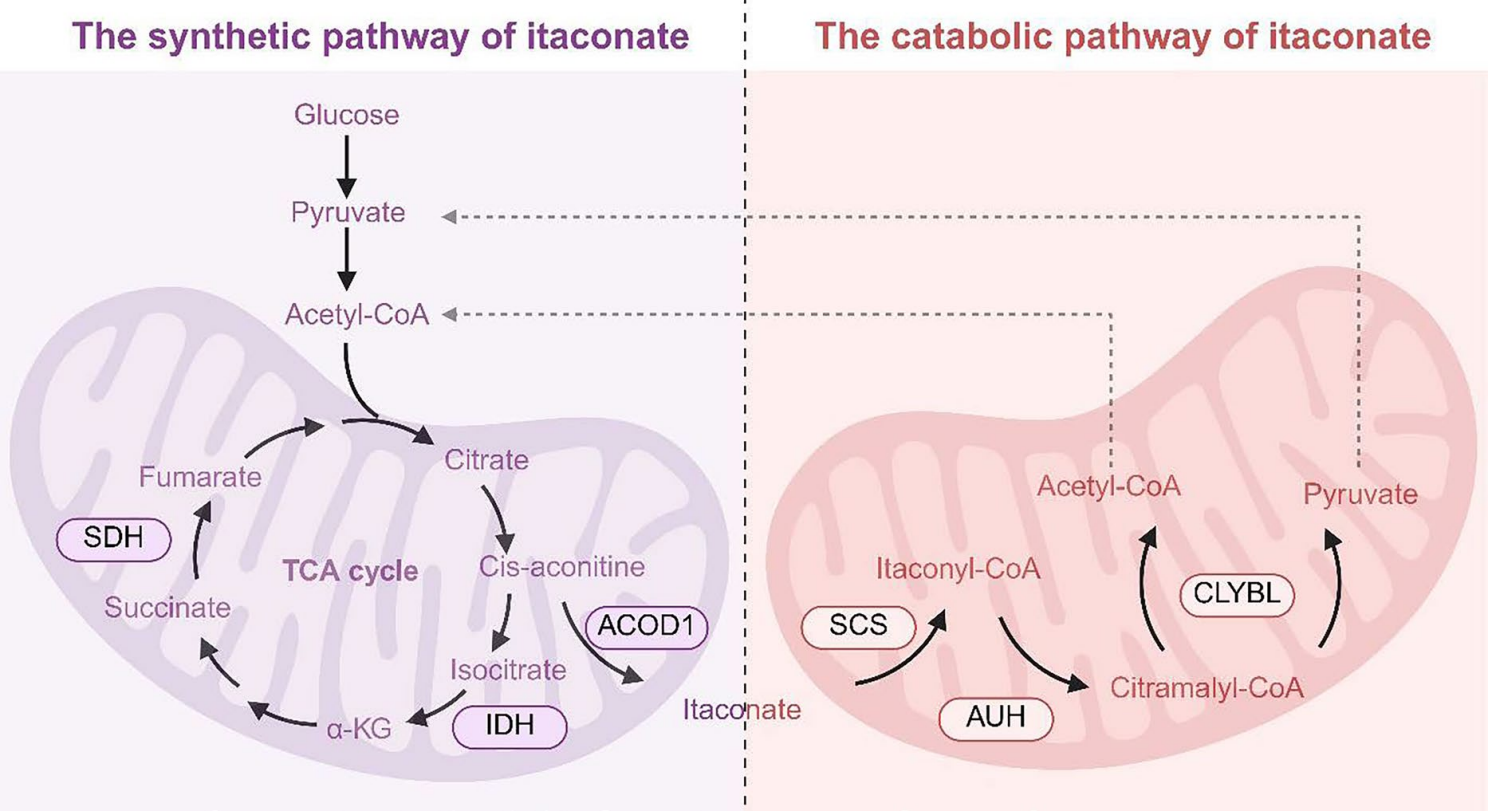
The synthetic and catabolic pathways of ITA. ITA is synthesized in the mitochondria of immune cells, such as macrophages. Its production is primarily catalyzed by the enzyme ACOD1, which diverts *cis*-aconitate from the TCA cycle. The catabolism of ITA involves a three-step enzymatic process within the mitochondria of ITA-degrading cells: it is first activated to itaconyl‑CoA, then isomerized to citramalyl-CoA by AUH, and finally cleaved into acetyl-CoA and pyruvate by CLYBL. This degradation pathway integrates ITA-derived carbon back into central metabolism. Schematics were created with BioRender.com

In contrast to the well-defined role of ACOD1 in the biosynthesis of ITA, the catabolic pathways of ITA remain inadequately characterized, and their physiological implications are still not well elucidated. It has been demonstrated, however, that in degrader cells for ITA, this reaction takes place within the mitochondria via three chemical steps, beginning with succinyl-CoA synthetase (SCS), which converts ITA to itaconyl-CoA: the subsequent enzyme is methylglutaconyl-CoA hydratase (AUH) converting itaconyl-CoA to citramalyl-CoA in a reorganization reaction. Lastly, citrate lyase subunit beta-like (CLYBL) breaks down citramalyl-CoA to pyruvate and acetyl-CoA [43] (Table 1). Currently, in mammals, these three enzymes that degrade ITA are primarily expressed in liver cells; it remains unclear whether other cell types express them under specific conditions. A schematic overview of both the anabolic and catabolic pathways of ITA is provided in Fig. 3. *In vivo*, ITA is rapidly metabolized to generate acetyl-CoA, mesaconate, and citramalate [44]. ITA administration also influences branched-chain amino acid metabolism and succinate levels, indicating a functional impact on SDH and methylmalonyl-CoA mutase (MCM) activity [44]. In the future, the metabolic and kinetic characteristics of ITA at both intracellular and *in vivo* levels need to be further elucidated to achieve a more comprehensive understanding of its regulatory functions in the bidirectional host-bacteria interaction across different spatiotemporal stages.

**Table 1:** Enzymes involved in ITA biosynthesis and catabolism in mammals

Enzyme abbreviations	Full name of the enzyme	Crystal structure (human)	Primary biological functions	Majorly expressed cell types	References
ACOD1 / IRG1/ CAD	Aconitate decarboxylase 1/Immuneresponsive gene 1 protein/*Cis*-aconitate decarboxylase		Catalyzing the decarboxylation of *cis*-aconitate, an intermediate in the TCA cycle, to form ITA	Myeloid cells, macrophages, neutrophils and monocyte-derived dendritic cells, cancerous epithelial cells	[3, 5, 26, 45–49]
SCS	Succinyl-CoA synthetase		Converting ITA to itaconyl-CoA	Liver cells	[43, 50, 51]
AUH	Methylglutaconyl-CoA hydratase		Converting itaconyl-CoA to citramalyl-CoA in a reorganization reaction	Liver cells	[43, 50–52]
CLYBL	Citrate lyase subunit beta-like		Breaking down citramalyl-CoA to pyruvate and acetyl-CoA	Liver cells	[43, 50, 51]

The key enzyme information related to ITA metabolism includes their abbreviations, full names, crystal structures, primary cell types expressing them, and supporting references. The crystal structure diagram is from the Protein Data Bank (PDB)

### Derivatives and isomers of ITA: tools and insights

The production of endogenous ITA is regulated by enzymes and induced by inflammatory stimuli or pathogenic bacteria infections; however, due to the polar nature of ITA, exogenous ITA has difficulty entering cells to exert its effects [53]. This property allows researchers to modify ITA by altering its polarity to create ITA derivatives for targeted delivery or as alternatives [53]. In the context of host-bacterial interactions-where ITA mediates bidirectional immune regulation in an “offense and defense” manner-these derivatives serve not only as tools to mimic ITA functions but also as probes to dissect its underlying molecular mechanisms.

Commonly used analogues include 4-Octyl Itaconate (4-OI), Dimethyl Itaconate (DMI), and 4-Ethyl Itaconate (Monoethyl itaconate, 4-EI): Only 4-OI can be hydrolyzed in a cell to regenerate active ITA. Research has shown that these alternatives do not have the same effects as natural ITA [24, 54, 55]. In 4-OI, the ester group is positioned distally to the double bond, which reduces its reactivity toward thiols and allows intracellular hydrolysis to release ITA, thereby mimicking its physiological activity [24, 54, 55]. Due to its ability to be hydrolyzed intracellularly to ITA and the stability conferred by its long carbon chain against esterase-mediated cleavage, enabling persistent modification of target proteins, which is a bioorthogonal probe named Itaconate-alkyne (ITalk) was developed for chemical proteomics studies. The covalent modification of proteins by ITA is termed “itaconation” [27]. Homocysitaconate is a newly identified metabolite formed from homocysteine and ITA through the action of S-adenosyl-L-homocysteine hydrolase; it exerts anti-inflammatory effects by targeting methionyl-tRNA synthetase [56]. Two isomers of ITA, mesaconate and citraconate, have also been reported to possess immunomodulatory functions similar to those of ITA [26, 57]; however, mesaconate does not affect SDH activity or the TCA cycle [26]. While ITA can serve as a metabolic precursor for the production of mesaconate [26, 57], it remains unclear whether mesaconate and citraconate can be converted into endogenous ITA within cells or whether they can exert antibacterial effects similar to those of ITA (Table 2).

**Table 2:** The structure of ITA derivatives, isomers and the metabolic relationship with ITA

Molecule	Formula	Structures	Metabolic relationship with ITA	References
ITA	C_5_H_6_O_4_		–	–
4-OI	C_13_H_22_O_4_		Converted to ITA	[24, 54, 55]
DMI	C_7_H_10_O_4_		Cannot be converted to ITA	[54, 58]
4-EI	C_7_H_10_O_4_		Cannot be converted to ITA	[54]
ITalk	C_13_H_18_O_4_		Cannot be converted to ITA	[27]
Homocysitaconate	C_9_H_15_NO_6_S		Homocysitaconate is formed from homocysteine and ITA through a reaction catalyzed by S-adenosyl-L-homocysteine hydrolase	[56]
Mesaconate	C_5_H_6_O_4_		A natural isomer of ITA. In macrophages, ITA serves as a metabolic precursor for the production of mesaconate	[57, 59]
Citraconate	C_5_H_6_O_4_		A potential ACOD1 inhibitor that binds competitively to inhibit ITA production	[57]

The structures of ITA, its isomers, and its derivatives, as well as their metabolic relationships with intracellular ITA

## ITA exerts antibacterial effects as a metabolic messenger

### ITA directly suppresses pathogenic bacteria: covalently modifies key metabolic enzymes

In the “offense and defense” interaction between host and bacteria, ITA serves as a crucial metabolic weapon deployed by the host. First and foremost, ITA exerts antibiotic activity against pathogenic bacteria by inactivating essential enzymes or pathways in these pathogenic bacteria. ITA regulates metabolic and immune responses by inducing “itaconation” of cysteine residues in proteins, or “itaconylation” of lysine residues in proteins following its metabolism into itaconyl-CoA [13, 27, 60].

The glyoxylate cycle is a classic target of ITA. In *Pseudomonas aeruginosa* (*P. aeruginosa*), ITA competitively inhibits isocitrate lyase (ICL) due to structural similarity with its substrate, isocitrate. By binding to the active site, ITA blocks the glyoxylate cycle, impairing energy metabolism and biofilm formation, ultimately suppressing bacterial proliferation under glucose-deficient conditions [17]. For *M. tuberculosis*, which is similarly susceptible: ITA covalently modifies Cys191 of ICL1 and Cys215 of ICL2, inhibiting their activity and stalling bacterial growth [17]. In *Vibrio cholerae* (*V. cholerae*) growth inhibition was observed in cultures grown on short chain fatty acids due to interference by ITA with the activity of isocitrate lyase (AceA) possibly methylisocitrate lyase (PrpB), which inhibits the glyoxylate cycle and/or methylisocitrate pathway, which are required in fatty acid metabolism [61].

Glycolysis and purine biosynthesis are also major ITA targets. ITA dose-dependently targets aldolase (Ald) and IMP dehydrogenase, disrupting both glycolysis and purine biosynthesis [62]; its activated form, itaconyl-CoA, irreversibly inhibits MCM, disrupting propionate metabolism essential for *M. tuberculosis* growth [18]. In *Salmonella enterica* serovar Typhimurium (*S.* Typhimurium), itaconation inhibits multiple enzymes in the *de novo* purine synthesis pathway (e.g., PurF, GuaA, GuaB, GuaC), restricting bacterial replication [63]. In* S. aureus*, ITA not only constrains glycolysis but also modulates the expression of stress response regulators (e.g., *glnR*, *kdpD*, *kdpE*, *sigB*), leading to dysregulation in acid and oxidative stress responses and protein homeostasis [19, 20].

Importantly, competition assays using bioorthogonal probes such as C3A reveal pathogen-specific target profiles, indicating that the antibacterial mechanisms of ITA are context-dependent and may vary across species [63]. Furthermore, current studies have largely focused on itaconation; the role of itaconylation in pathogenic bacteria remains to be confirmed.

### ITA indirectly restricts pathogenic bacteria: regulating cellular signal transduction

In addition to their direct antibacterial and metabolic-disrupting effects, ITA can also help defend against pathogenic bacteria by modulating the host immune system. In non-immune cells, however, ITA enhances innate antimicrobial immune function upon cellular uptake.

The regulation of immune cells by ITA is primarily reflected in the host’s defense against intracellular bacterial infections. During *Salmonella* infection, TFEB is activated, leading to the transcriptional upregulation of *ACOD1* expression and increased production of mitochondrial ITA [64]. Subsequently, under the action of the protein leucine-rich repeat kinase 2 (LRRK2) and Rab32 complex, mitochondria expressing ACOD1 are localized to *Salmonella* vesicles, thereby facilitating the targeted delivery of ITA [65, 66]. Through the action of MCT1 and MCT4 on *Salmonella* vesicles, ITA is transported into the vesicles, exerting metabolic stress on the bacteria [67]. Notably, *Salmonella* vesicles retain an acidic pH (around 5.0) with an ITA accumulation of about 5–6 mM in line with potent antimicrobial activities at a low-pH with reduced minimal inhibitory concentration at acidic conditions [65]. This intracellular environment enhances the ability of ITA to inhibit the growth of intracellular pathogenic bacteria, particularly *S*. Typhimurium. The rapid response of macrophages to *Salmonella* and the targeted delivery of ITA enable host cells to establish a rapid and effective mechanism to suppress the proliferation of intracellular *Salmonella*. Additionally, ITA enhances macrophage clearance of *S.* Typhimurium by promoting lysosomal biogenesis through alkylation of TFEB [68].

The immunosuppressive properties of ITA are not limited to a direct effect on immune cells but also include actions within liver cells, the transporter solute carrier family 13 member 3 (SLC13A3) mediates ITA uptake, leading to activation of TFEB-mediated lysosomal biogenesis, thus enhancing host defence responses [30]. During *P. aeruginosa* airway infection, secreted ITA activates OXGR1 on lung epithelial cells, enhancing mucociliary clearance and mucin secretion to expedite bacterial removal [27].

In summary, ITA exhibits broad-spectrum antibacterial activity against both Gram-negative (e.g., *S*. Typhimurium) and Gram-positive (e.g., *S. aureus*) bacteria. It directly inhibits bacterial growth by disrupting metabolic pathways and also coordinates immune cells or enhances non-immune cell antimicrobial functions. The mechanisms underlying ITA-mediated inhibition of bacterial infections are summarized in Table 3. These findings clearly demonstrate the multifaceted role of ITA as an “offensive” molecule in host-bacteria interaction; bacterial “defensive” strategies against ITA will be discussed in subsequent sections.

**Table 3:** The mechanism by which ITA inhibits pathogenic bacterial infections

Bacteria	Gram	Mechanism	Disease	References
*S.* Typhimurium	Negative	(1) Under the influence of Rab32 and LRRK2, ITA localizes to *Salmonella* vesicles and is transported into them via MCT1 and MCT4, where it directly exerts its bacteriostatic effect; (2) Inhibiting the *de novo* purine synthesis pathway, thereby limiting bacterial growth; (3) Promoting lysosomal biogenesis and bacterial clearance through alkylation of TFEB at Cys212	*S.* Typhimurium infections (enteritis, typhoid, etc.)	[63, 65–68]
*P. aeruginosa*	Negative	(1) Inhibiting the glyoxylate cycle; (2) Activating OXGR1 to achieve mucociliary clearance in lung epithelial cells; (3) Suppressing LPS assembly and selects for low-virulence bacterial strains	Cystic fibrosis infections	[34, 69]
*Klebsiella pneumoniae* (*K. pneumoniae*)	Negative	Inhibiting the kinase activity of SYK, thereby suppressing hypervirulent *Klebsiella pneumonia*-mediated inflammation and macrophage death	Pneumonia; Urinary tract infection; Sepsis	[70]
*S. aureus*	Positive	(1) Promoting phagocytosis by macrophages; (2) Reducing bacterial energy metabolism and arginine biosynthesis; (3) Inducing oxidative stress and acid stress	Skin infections; Pneumonia; Sepsis	[71–73]
*M. tuberculosis*	Acid‑fast (atypical Gram‑positive cell wall)	Inhibiting glyoxylate cycle, glycolysis, purine metabolic pathways, and propionate metabolism	Tuberculosis	[17, 18, 62]

Based on Gram staining and other properties, the table summarizes the mechanisms by which ITA inhibits common pathogenic bacteria, along with relevant diseases and references

## Mechanisms of pathogenic bacteria degradation of ITA and pathogenic bacteria adaptation pathways to ITA stimulation

### Direct degradation and utilization of ITA by pathogenic bacteria: enzymatic degradation of ITA mediated by the IcT-IcH-CcL cascade

Although ITA exerts broad-spectrum antimicrobial activity through multiple direct and indirect mechanisms, some pathogenic bacteria have evolved specialized enzymatic systems to degrade ITA, thereby alleviating the metabolic stress imposed by ITA. The bacterial metabolism of ITA typically involves three enzymatic steps, namely the IcT-IcH-CcL enzymatic cascade. Firstly, the itaconate coenzyme A-transferase (IcT) transfers a CoA moiety of succinyl-CoA to ITA, which yields itaconyl-CoA and succinate. Itaconyl-CoA is then hydrated by itaconyl-CoA hydratase (IcH), yielding citramalyl-CoA whose conversion to acetyl-CoA and pyruvate is completed by citramalyl-CoA lyase (CcL) [45, 74–77]. Furthermore, in response to ITA stimulation, ICL in the glyoxylate cycle is inhibited, leading to the accumulation of isocitrate. This, in turn, stimulates RipR-mediated upregulation of the transcription of *ict*, *ich*, and *ccl*, enabling the pathogenic bacteria to rapidly respond by degrading ITA [76].

Genetic organization of the corresponding enzymes varies across bacterial species. In *Yersinia pestis* (*Y. pestis*), the corresponding genes are *ripA* (for IcT), *ripB* (encoding IcH) and *ripC* (encoding CcL). Homologous three-gene clusters are present in *Streptococcus enterocolitica*, *Burkholderia nosocomialis*, *Bacillus cereus*, *Mycobacterium ulcerans*, *Mycoplasma synoviae*, and *Legionella longbeachae*. In contrast, *Brucella* and *Bordetella* species exhibit a more complex genomic arrangement resembling that of *P. aeruginosa*, with the three core genes co-localized alongside three additional open reading frames [74, 75]. *M. tuberculosis* encodes functional homologs involved in ITA degradation: Rv2503c and Rv3272 display IcT activity; Rv2499c and Rv3389c exhibit IcH activity; and Rv2498c functions as a bifunctional CcL enzyme that participates in both L-leucine and ITA catabolism [77]. These findings suggest that metabolic degradation of ITA by pathogenic bacteria may be widespread, and enzymes degrading ITA may also perform functions beyond enzymatic degradation. This reflects the complex adaptive strategies evolved by pathogenic bacteria and hosts through prolonged antagonism. Future identification of IcT, IcH, and CcL isoenzymes in other pathogenic bacteria and investigation of their functional activities will enhance our understanding of ITA-mediated host-microbe interactions. In the case of *Salmonella*, certain serotypes, such as *S*. Typhimurium LT2, lack a complete set of classical degradation genes. Instead, these bacteria carry open reading frames (e.g., *stm3117*, *stm3118*, and *stm3119*) that exhibit 77%, 87%, and 72% amino acid identity to RipA, RipB, and RipC from *Y. pestis*, respectively, which may contribute to the metabolism of ITA. Based on previous studies, the standard genomic annotations currently classify STM3117 as a lactoylglutathione lyase, STM3118 as an acetyl-CoA hydrolase/transferase, and STM3119 as a member of the monoamine oxidase family with putative dehydratase activity [78]. Although these genes have been tentatively designated as *ict*, *ich*, and *ccl* based on sequence homology, their precise biochemical roles require further functional validation [78]. These discrepancies highlight the need for direct biochemical characterization, including enzyme activity assays, substrate specificity tests, and metabolic profiling to conclusively determine whether these gene products are functionally involved in ITA degradation in *Salmonella*. Further evidence is needed to determine whether pathogens employ a widespread IcT-IcH-CcL cascade-mediated degradation strategy to counteract ITA.

### Adaptive pathways of pathogenic bacteria in response to ITA stimulation

Beyond direct enzymatic degradation of ITA, numerous pathogenic bacteria have developed sophisticated adaptive strategies to counteract its antimicrobial effects. These adaptive mechanisms can be summarized in two aspects: first, numerous pathogenic bacteria evade targeted killing by ITA by interfering with signal transduction in host immune cells; second, pathogenic bacteria undergo metabolic reprogramming in response to ITA stimulation, shifting from highly toxic acute infections to low-toxicity chronic infections.

An example of pathogenic bacteria interfering with immune cell signal transduction to adapt to metabolic stress induced by ITA is *Salmonella*. In *Salmonella*, effector proteins such as SopD2 and GtgE facilitate the inactivation or degradation of the small GTPase Rab32, thereby disrupting the targeted delivery of ITA to bacteria-containing vesicles on the host [66]. As a classic intracellular bacterium, *Salmonella* disrupts the protein complexes responsible for the targeted delivery of ITA by secreting effector molecules, thereby ensuring its survival and infection. *L. monocytogenes*, which is also an intracellular bacterium, may employ similar mechanisms to resist targeted delivery of ITA; however, further research is needed to support this hypothesis.

Adaptation to ITA stimulation through metabolic reprogramming, altering survival strategies, is primarily observed in *P. aeruginosa* and *S. aureus*. Substrate-binding protein, IctP (PA0884), designated as IctPQM, is responsible for the transport and uptake of ITA in *P. aeruginosa* [79]. *P. aeruginosa* undergoes extensive metabolic reprogramming in response to ITA, characterized by downregulation of LPS synthesis and upregulation of exopolysaccharide (EPS) production. ITA-adapted strains frequently acquire mutations in lipopolysaccharide assembly protein LptD (*lptD*), which encodes an LPS transport protein, further promoting ITA assimilation and enhanced biofilm formation [69, 80]. Additionally, ITA reduces acetylation of the RNA chaperone CspC, strengthening its binding to *rsaL* mRNA and resulting in upregulation of the quorum sensing system [81]. These changes promoted biofilm formation and activated quorum sensing, thereby increasing the propensity for chronic infection. Rising EPS production increases ITA made by both airway and systemic myeloid cells, creating a positive feedforward loop that continues the infection. Moreover, during airway infection, ITA promotes ketogenesis, inducing metabolic stress that selects for *P. aeruginosa* variants with reduced LPS, thereby facilitating host coexistence [74]. In addition to reprogramming toward EPS production and biofilm formation, *P. aeruginosa* also utilizes S-itaconated RpoN to enhance glucose catabolism and the Entner-Doudoroff pathway, thereby improving its survival [82]*.* Similar to *P. aeruginosa*, *S. aureus* also undergoes adaptive changes, manifested by reduced glycolysis, as well as inhibition of pyruvate metabolism and arginine biosynthesis; carbon flux is redirected toward EPS production and biofilm formation to sustain the infection [19, 73].

**Fig. 4:**
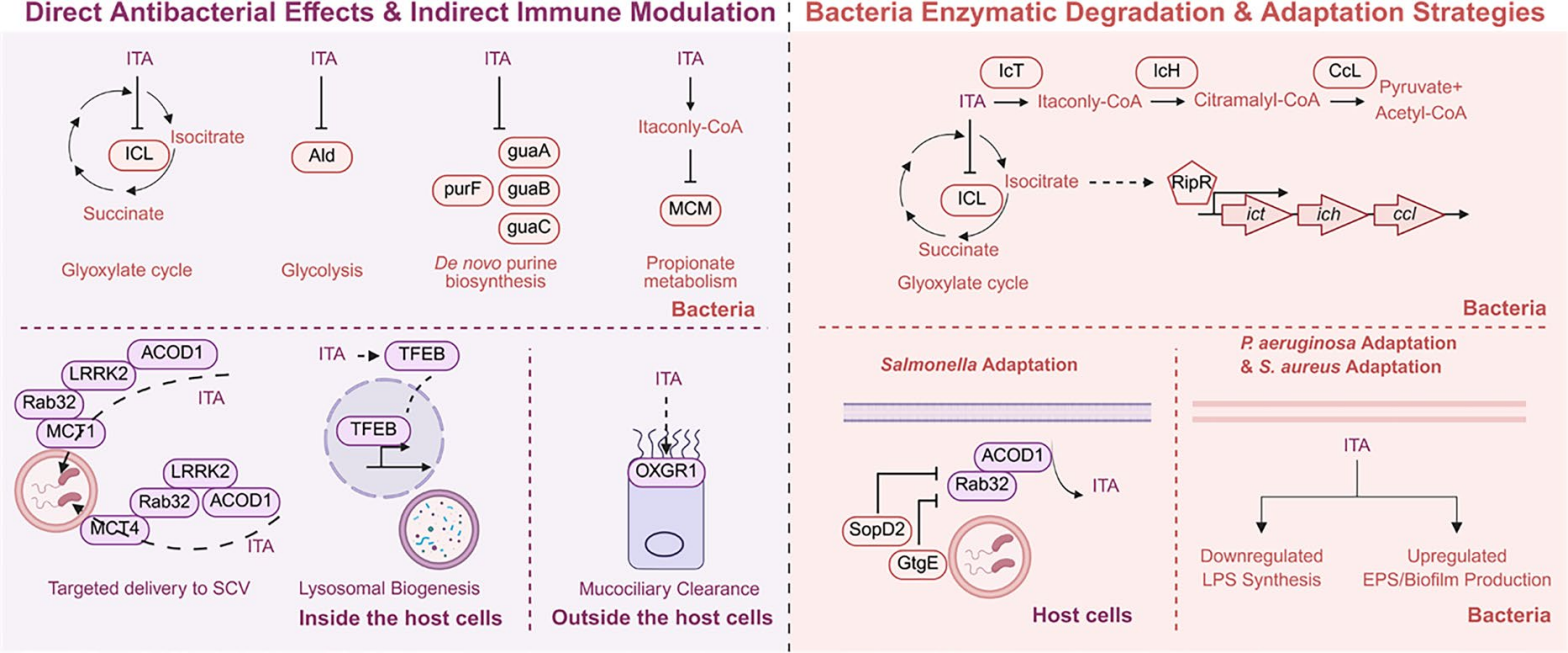
Mechanisms of ITA-mediated pathogenic bacteria inhibition (direct and indirect) and mechanisms by which pathogenic bacteria degrade or adapt to ITA. The primary direct mechanisms by which ITA inhibits pathogenic bacteria involve inhibition of the bacterial glyoxylate cycle, glycolysis, *de novo* purine synthesis, and propionate metabolism. The indirect mechanisms of ITA inhibition include the targeted suppression of *Salmonella* through transport into the SCV via MCT1/MCT4 under the action of Rab32- and LRRK2-mediated protein complex, as well as the induction of TFEB-mediated lysosomal biogenesis and the activation of OXGR1-mediated mucociliary clearance. Pathogenic bacteria, however, have evolved mechanisms to counteract ITA stress, including the IcT-IcH-CcL cascade reaction that directly degrades ITA, as well as various adaptive pathways that drive persistent infection. Schematics were created with BioRender.com

In summary, some pathogenic bacteria directly degrade ITA via the IcT-IcH-CcL cascade and convert it into their own carbon source. In parallel, they adapt by interfering with the targeted delivery of ITA within host cells or through metabolic reprogramming. These strategies ensure their survival and persistent infection (Table 4). As described earlier, ITA suppresses or eradicates pathogenic bacteria by directly inhibiting key metabolic pathways and coordinating host immune responses. As a metabolic messenger, ITA plays a central role in the “offense-defense” dynamics between host and pathogenic bacteria (Fig. 4). Further elucidation of these bidirectional resistance mechanisms may improve our understanding of host-bacterial interactions and inform the development of more targeted strategies to enhance bacterial clearance.

**Table 4:** Mechanisms of catabolism or adaptation of ITA by various common pathogenic bacteria

Bacteria	Gram	Mechanism	Diseases	References
*S.* Typhimurium	Negative	1. IcT-IcH-CcL-mediated cascade enzymatic degradation (require further functional validation), isocitrate activates RipR-mediated transcription of *ict*, *ich*, and *ccl*; 2. Secreted effectors degrade Rab32 to evade targeted delivery by intracellular ITA	*Salmonella* infections (such as enteritis, typhoid)	[65, 76]
*P. aeruginosa*	Negative	1. IcT-IcH-CcL-mediated cascade enzymatic degradation; 2. ITA drives chronic infection through CspC-RsaL-enhanced quorum sensing; 3. S-itaconated RpoN enhances glucose catabolism and the Entner-Doudoroff pathway; 4. *LptD* mutations promote EPS and biofilm formation	Hospital-acquired infections; Cystic fibrosis infections	[69, 75, 81, 82]
*Y. pestis*	Negative	IcT-IcH-CcL-mediated cascade enzymatic degradation	Bubonic plague	[75]
*S. aureus*	Positive	1. Inhibition of pyruvate metabolism and arginine biosynthesis; 2. Carbon flux is redirected toward EPS production and biofilm formation to sustain the infection	Skin infections; Pneumonia; Sepsis	[19, 73]
*M. tuberculosis*	Acid‑fast (atypical Gram‑positive cell wall)	IcT-IcH-CcL-mediated cascade enzymatic degradation	Tuberculosis	[77]

Based on Gram staining and different characteristics, the mechanisms by which common pathogenic bacteria metabolize ITA and their adaptive mechanisms are categorized in the table, along with a list of related diseases and references

## Spatiotemporal specificity of ITA in bacterial infection: from early antibacterial defense to late immunomodulation

While pathogenic bacteria have evolved strategies to resist ITA-mediated inhibition, the role of ITA in host immunity is not static-it varies with the stage of infection, showing distinct spatiotemporal specificity and cellular heterogeneity. In this section, we discuss the spatiotemporal specificity of ITA, distinguishing its early antibacterial functions from its later immunomodulatory roles.

### Early phase: direct and indirect antibacterial actions

ITA acts as a metabolic messenger that rapidly responds to pathogen infection and restricts disease progression at an early stage. Its early antibacterial effects exhibit spatiotemporal specificity via direct and parallel indirect mechanisms. Directly, ITA rapidly targets evolutionarily conserved metabolic hubs in pathogens (e.g., glyoxylate cycle, glycolysis, purine synthesis, and propionate metabolism) through itaconation and itaconylation, suppressing bacterial energy supply and biosynthesis at the initial infection stage [17, 18, 62, 63]. Indirectly, ITA harnesses host membrane transporters and organelle-directed delivery to early activate lysosomal biogenesis and trigger mucociliary clearance in non-immune cells (e.g., lung epithelial cells), thereby synergistically enhancing innate immune defense [27, 30, 66–68]. Together, these pathways form a cascade network of “metabolic interference-immune synergy” across the spatiotemporal dimensions of infection.

### Late phase: resolution of inflammation and adaptive immunity modulation

As pathogenic bacteria infection becomes contained, the host’s inflammatory response needs to gradually resolve to mitigate excessive immune reactions. ITA primarily exerts anti-inflammatory effects during the late stage of infection. For example, in the case of hypervirulent *Klebsiella pneumoniae* infection, ITA targets the modification of the Cys593 residue on spleen tyrosine kinase (SYK) and thereby attenuates uncontrolled inflammation [70].

**Fig. 5:**
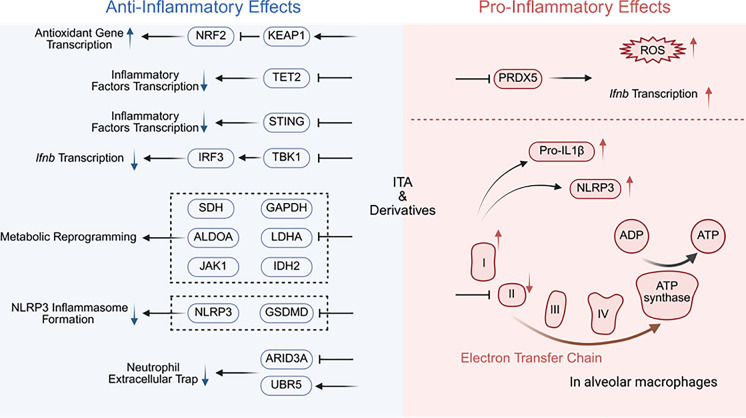
The dual pro-inflammatory and anti-inflammatory effects of ITA. ITA has been reported to exert both pro-inflammatory and anti-inflammatory effects. Its anti-inflammatory mechanisms primarily include: (1) specifically inhibiting KEAP1 to activate NRF2 and promote the expression of oxidative stress-related genes; (2) inhibiting TET2-mediated expression of pro-inflammatory genes; (3) inhibiting the STING signaling pathway to suppress the expression of pro-inflammatory genes; (4) inhibiting TBK1 to suppress IRF3-mediated transcription of IFN-β; (5) inhibiting metabolic enzymes such as SDH to drive metabolic reprogramming; (6) inhibiting GSDMD and NLRP3 to suppress macrophage death; (7) inhibiting ARID3A and alkylating UBR5 to inhibit the formation of neutrophil extracellular traps. Its pro-inflammatory pathways primarily include: (1) targeting and inhibiting PRDX5 to increase ROS and promote *Ifnb* transcription; (2) inhibiting the electron transport chain in alveolar macrophages to promote inflammation. The specific pro-inflammatory or anti-inflammatory effects of ITA are determined by cellular heterogeneity and the specific stage of the immune response

In summary, ITA and its derivatives limit the secretion of inflammatory factors through multiple mechanisms, primarily including the following: (1) target inhibition of KEAP1, thereby activating NRF2 to promote the transcription of antioxidant genes [24]; (2) target inhibition of TET2 activity, thereby suppressing the levels of inflammatory factors and chemokines such as *il6* and *ccl5* [83]; (3) target inhibition of STING to limit its activation and suppress the production of downstream inflammatory factors [84, 85]; (4) targeting TBK1 to inhibit type I interferon production [86, 87]; (5) targeting NLR family pyrin domain-containing 3 (NLRP3) and gasdermin D (GSDMD) to inhibit NLRP3 inflammasome formation [88, 89]; (6) targeting enzymes such as SDH, glyceraldehyde-3-phosphate dehydrogenase (GAPDH), fructose-bisphosphate aldolase A(ALDOA), lactate dehydrogenase A (LDHA), janus kinase 1(JAK1) and IDH2 to drive metabolic reprogramming in macrophages [23, 28, 40, 55, 90]; (7) targeting the E3 ubiquitin ligase component N-recognin 5 (UBR5) and AT-rich interactive domain-containing protein 3A (ARID3A) protein inhibits the formation of neutrophil extracellular traps [91, 92]. These changes guide immune cells, including macrophages, neutrophils, and others, toward an anti-inflammatory state to limit excessive inflammation. However, ITA does not simply always suppress inflammation; at times, it can also promote inflammation. A typical example is that while ITA exerts an anti-inflammatory effect in monocytes and macrophages, it promotes inflammation in alveolar resident macrophages, illustrating the dual role of ITA in inflammation regulation. In addition, ITA can increase reactive oxygen species (ROS) levels by targeting PRDX5 and promote the production of type I interferons (Fig. 5). Therefore, ITA should be regarded as an immunoregulatory factor, and its specific functions should be analyzed in conjunction with the spatial specificity and cellular heterogeneity of the infection site under different infection conditions and at different stages of infection.

In the later stages of a pathogenic bacteria infection, the immune system develops adaptive immunity. This “immune memory” enables the host to mount a rapid immune response against the same pathogenic bacteria upon re-exposure. Current reports on ITA-mediated adaptive immunity have primarily focused on research into the tumor microenvironment. ITA inhibits the biosynthesis of aspartic acid and serine/glycine in CD8+ T cells and induces their exhaustion [33, 48]; it also suppresses Th17 cell differentiation and promotes Treg cell differentiation [58]. However, it remains to be further investigated whether these effects are also present in “immune memory” during the later stages of infection with pathogenic bacteria. In addition, “immune memory” can also occur in innate immune cells; this is known as “trained immunity” [93, 94]. Currently, research on the role of ITA in trained immunity remains limited. It is known that DMI can enhance trained immunity via the glutathione synthesis pathway [95], but it inhibits β-glucan-induced trained immunity [96]. Although DMI confers the ability to combat *S. aureus* infection on monocytes through trained immunity, this effect cannot be equated with that of endogenous ITA. There are relatively few reports on the effects of ITA in training the immune system to resist pathogenic bacteria infections, and how this reshaping of the innate immune system leads to antimicrobial activity is also a topic worthy of attention. In summary, ITA plays different roles at various stages of pathogenic bacteria infection, and its impact on the development of the immune system varies depending on whether it involves innate or adaptive immunity. Further research should focus on the spatiotemporal specificity of ITA to elucidate its specific mechanisms.

### Special homeostatic reshaping: spatiotemporal specificity of ITA in gut microbiota regulation

As an organ that naturally harbors commensal microbes, the gut has a complex process of shaping immune homeostasis [97]. This means that the function of ITA in the gut not only requires inhibiting pathogenic bacteria during the early stage of infection and suppressing excessive inflammation during the late stage of infection, but also necessitates maintaining microbial balance during infection or promoting the restoration of microbial balance after infection, rendering its spatiotemporal specificity even more pronounced^111^.

In infectious settings, ITA helps restore intestinal microbiota balance in mice following infection with *Listeria monocytogenes* or *Citrobacter rodentium* [61]. Under non-infectious conditions, *Acod1*-knockout mice display significantly altered abundances of two Ruminococcaceae species and one Clostridiales species compared to wild-type mice, indicating that ITA also contributes to microbiota composition at steady state [61]. However, under a high-fat diet, oral administration of ITA exacerbates gut dysbiosis and obesogenic responses-an effect abrogated in *Acod1*-knockout mice [98]. These contrasting outcomes demonstrate that ITA's regulation of the gut microbiota is not fixed but rather highly dependent on disease context, dietary background, and the local metabolic environment of the gut-a pattern consistent with the spatiotemporal specificity observed in other infection stages. Second, ITA can be used for anti-inflammatory purposes following infectious diseases or for inflammatory regulation.

Current research on ITA in gut microbiota homeostasis primarily focuses on overall community structure. Whether ITA directly modifies key metabolic enzymes of probiotics to promote their growth and colonization remains an open question. Notably, a homolog of ACOD1, designated bsIRG1, has been identified in *Bacillus subtilis* [99], suggesting that some probiotics may intrinsically possess the ability to produce ITA. Nevertheless, direct experimental evidence for bacterially derived ITA modulating host immunity during human or other animal infections is still lacking. Therefore, exploring the gut microbiota as a potential endogenous source of ITA and elucidating its spatiotemporal roles in local immunity and microbial homeostasis during different stages of infection may represent an important direction for future research.

## Application prospects and challenges of ITA

Based on the bidirectional regulatory mechanisms of ITA in host-bacteria interactions and its spatiotemporal characteristics during infection, ITA-related pathways and compounds may provide useful insights for developing adjunctive or antibiotic-alternative strategies for bacterial disease control. However, their translational application remains preliminary and faces several important limitations. This section discusses the potential prospects and challenges of ITA-related interventions.

### Prospects for clinical application

As the functions and mechanisms of ITA at different stages of infection and against different pathogens are gradually elucidated, ITA-related pathways have attracted attention as potential targets for immunometabolic modulation. However, current evidence is mainly derived from in vitro studies, animal models, or pharmacological derivatives such as 4-OI and DMI. Therefore, the clinical efficacy, safety, dosage, pharmacokinetics, and context-specific effects of ITA-related interventions remain to be systematically evaluated before clinical translation.

First, ITA-related interventions may have potential as adjunctive or antibiotic-sparing strategies for bacterial infections, but they should not yet be considered established substitutes for antibiotics. For example, patients with bacterial endophthalmitis have elevated levels of ITA in their vitreous humor. In a mouse model of bacterial uveitis, intraocular administration of DMI or 4-OI was reported to reduce inflammation and bacterial load while preserving retinal structure and visual function [100]. These findings suggest a potential therapeutic direction, but further validation is required before ITA-related interventions can be considered for clinical application as adjunctive or antibiotic-sparing approaches. Moreover, the combination of vancomycin and 4-OI can further reduce bacterial load [100]. This provides a new approach for treating bacterial infections, although more clinical evidence is still needed to validate its safety and efficacy. Second, ITA and its derivatives may have potential for modulating inflammation after infectious diseases or in selected non-infectious inflammatory conditions, although their efficacy, safety, dosage, and context-specific effects require further investigation [101]. Currently, clinical evidence regarding ITA is limited, and further clinical trials are needed to elucidate its effects. Additionally, since ITA and its derivatives exhibit differences in electrophilic properties and physicochemical characteristics, these factors should be carefully considered in clinical trials.

### Applications in animal farming

In addition to its applications in human health, the application prospects in animal husbandry are also worthy of attention. On the one hand, ITA has a wide range of production sources and low cost, giving it the potential to be used as an organic acid additive in the feed industry. On the other hand, the prohibition of antibiotics in feed requires effective antibiotic alternatives to cope with pathogenic *E. coli* infections in piglets, *Salmonella* infections in poultry, and so on^108^, ^109^. These characteristics of ITA are highly consistent with the goals of animal husbandry.

Currently, reports on ITA and its derivatives in animal husbandry are still limited, confined to only a few species, and there is a lack of clear industry application standards. In broilers, ITA improves growth performance, modulates cecal microbiota, and alleviates oxidative stress [102, 103]. In laying hens, ITA maintains microbial balance and reduces oxidative stress [71]. The ITA derivative 4-OI also exhibits direct inhibitory effects against avian pathogenic *E. coli*, targeting the inhibition of biofilm formation and reducing its tolerance to acid–base and osmotic stress [104]. Future studies should more broadly validate the application potential of ITA in other species, such as dairy cows (e.g., rumen microbial balance and lactation performance) and aquatic animals (e.g., intestinal microbial balance and feed conversion ratio), to better understand the extensive role of ITA in animal husbandry [97, 105, 106]. In addition, exploring itaconate or itaconate-related compounds under non-infectious conditions may provide useful information on their broader biological functions beyond immune regulation. However, their potential use as feed additives remains speculative and requires systematic evaluation of efficacy, safety, dosage, metabolism, microbiome effects, and long-term outcomes in relevant animal models.

### Limitations in the application and future research directions of ITA

Although ITA exhibits the powerful application potential described above, its application still has certain limitations: (1) The lack of targeted delivery strategies. For example, when using ITA to clear *Salmonella* infection, ITA must be precisely delivered to *Salmonella*-containing vacuoles. Unlike endogenous ITA, which is supported by a complete set of defense mechanisms, exogenous ITA struggles to achieve such precise targeting. Molecular modification approaches for targeted delivery have shown some promise-for instance, replacing ITA with 4-OI improves permeability. However, the physicochemical properties and target sites of these two compounds are not identical, which may pose potential risks. Meanwhile, liposome-based targeted delivery strategies are currently limited to delivering ITA to specific host cells [107]; liposomes that target specific pathogens require further design and modification^110^. (2) The need for more precise combination treatment regimens to reduce pathogen resistance to ITA. Exposure to ITA pressure triggers a series of adaptive strategies in pathogens, which can lead to chronic infection and impede complete bacterial clearance. 3-phenylpropionic acid inhibits the transcription of *ict*, *ich*, and *ccl* [76], thereby suppressing *Salmonella*-mediated degradation of ITA and synergistically enhancing pathogen clearance. Future efforts should focus on evaluating more such regimens to strengthen strategies for the complete eradication of pathogens. (3) The need for precise control over the timing and route of ITA administration. ITA plays different roles at different stages of infectious diseases, and owing to the heterogeneity of the target cells, it can exert opposite effects on the regulation of inflammation. These characteristics necessitate precise therapeutic protocols for the clinical application of ITA to minimize risks.

## Conclusions

As ITA was discovered as a natural metabolite in animals and its extensive metabolic and immunomodulatory roles were revealed, it has established an important position bridging metabolism and immunity, highlighting its significant biological significance. Most studies have focused on the distinct roles of ITA in regulating inflammation, whereas the “bidirectional effects” of its antibacterial activity and bacterial resistance to ITA have rarely been reported. This review aims to fill the gap in the field of ITA-mediated host-bacteria interaction. This review first introduced the research progress and basic metabolic pathways of ITA. We then focused on elucidating and summarizing the mechanisms by which ITA exerts antibacterial effects, either directly or indirectly, as well as the mechanisms by which pathogenic bacteria directly metabolize ITA and convert it into their own carbon source, along with other adaptive pathways. By summarizing the ITA-mediated “offense and defense” framework, we have conducted an in-depth analysis from a bidirectional perspective of host-bacteria interaction, revealing the complex regulatory relationships of co-development, co-metabolism, and co-evolution between the host and bacteria. Based on this framework, we further highlighted the differences in ITA across various stages of infection, providing a molecular basis and targeted recommendations for the application of ITA-related therapies across different disease types and infection stages. As an organ naturally harboring symbiotic bacteria, the gut exhibits more complex mechanisms under various infection scenarios due to the presence of probiotics in its environment. Therefore, we specifically summarized the unique role of ITA in the gut and analyzed the potential of probiotics to provide non-host-derived ITA. Finally, the review focused on the potential and challenges of ITA-related therapies as alternative strategies to antibiotics in clinical and animal husbandry applications.

Several limitations of this review and of the current evidence should be acknowledged. First, many available findings are derived from specific cell types, animal models, bacterial species, or experimental conditions and should not be directly generalized to all host-bacteria interactions. Therefore, the biological effects and potential applications of ITA should be interpreted in a context-specific manner. Second, endogenous ITA and pharmacological derivatives such as 4-OI and DMI may differ in bioavailability, electrophilicity, target specificity, and biological effects. Third, this review mainly focuses on ITA-mediated host-bacterial interactions, particularly those involving pathogenic bacteria; interactions with other microorganisms, such as viruses and fungi, were beyond the main scope of this review and were not comprehensively discussed. In summary, the therapeutic or agricultural application of ITA-related interventions remains preliminary and requires further validation in well-controlled *in vivo*, clinical, and field studies, including assessments of dosage, safety, pharmacokinetics, microbiome effects, and long-term outcomes.

Future studies on ITA-related interventions may focus on: (1) mapping ITA-pathogenic bacteria-antibiotic interactions using proteomic and transcriptomic approaches; (2) designing ITA derivatives or analogues with improved pharmacological properties while minimizing potential adverse effects or resistance-related concerns; (3) developing targeted delivery systems to improve tissue specificity and infection-site exposure; (4) establishing diagnostic frameworks to identify infection contexts or patient populations that may be more likely to benefit from ITA-related adjunctive strategies. If supported by further mechanistic, preclinical, and clinical evidence, ITA-related approaches may eventually inform more precise and sustainable strategies for managing biofilm-associated and intracellular infections. Future studies integrating immunology, microbiology, structural biology, metabolomics, and translational models will be essential for clarifying how ITA participates in host-bacteria interactions and for determining whether ITA-related compounds can be safely and effectively developed as adjunctive strategies for bacterial disease management. Such studies may provide useful insights into host-bacterial interactions and inform the development of antibiotic-sparing or complementary approaches for managing bacterial infections, including those caused by multidrug-resistant pathogens.

## Data Availability

No datasets were generated or analysed during the current study.

## Abbreviations

4-EI: 4-Ethyl Itaconate
4-OI: 4-Octyl Itaconate
β-arrestin2: Arrestin beta 2
ABCG2: ATP-binding cassette transporter protein G2
AceA: Isocitrate lyase
ACOD1: Aconitate decarboxylase 1
*A. itaconicus*: *Aspergillus itaconicus*
Ald: Aldolase
ALDOA: Fructose-bisphosphate aldolase A
ARID3A: AT-rich interactive domain-containing protein 3A
*A. terreus*: *Aspergillus terreus*
ATF3: Activating transcription factor 3
AUH: Methylglutaconyl-CoA hydratase
CAD: Aconitate decarboxylase 1
CcL: Citramalyl-CoA lyase
C/EBPβ: CCAAT enhancer binding protein beta
CLYBL: Citrate lyase subunit beta-like
DMI: Dimethyl Itaconate
*E. coli*: *Escherichia coli*
EPS: Exopolysaccharide
GAPDH: Glyceraldehyde-3-phosphate dehydrogenase
GlnR: Transcriptional repressor GlnR
GSDMD: Gasdermin D
GuaA: GMP synthetase
GuaB: IMP dehydrogenase
GuaC: GMP reductase
HIF-1α: Hypoxia-inducible factor 1 subunit alpha
IcH: Itaconyl-CoA hydratase
ICL: Isocitrate lyase
IcT: Itaconate coenzyme A-transferase
IDH: Isocitrate dehydrogenase
IFNAR: Interferon alpha and beta receptor
IFNGR: Interferon gamma receptor
IFN-I: Type I interferons
IRF: Interferon regulatory factor
IRG1: Immune response gene 1
ITA: Itaconate
ITalk: Itaconate-alkyne
JAK1: Janus kinase 1
JUN: JUN proto-oncogene
KdpD: Sensor protein KdpD
KdpE: Transcriptional regulator KdpE
*K. pneumoniae*: *Klebsiella pneumoniae*
LPS: Lipopolysaccharides
*lptD*: Lipopolysaccharide assembly protein LptD
LRRK2: Leucine-rich repeat kinase 2
LTTR: LysR-type transcriptional regulator
MCM: Methylmalonyl-CoA mutase
MCT1: Monocarboxylate transporter 1
MCT4: Monocarboxylate transporter 4
*M. tuberculosis*: *Mycobacterium tuberculosis*
NF-κB: Nuclear factor kappa-B
NLRP3: NLR family pyrin domain containing 3
NRF2: NFE2 like bZIP transcription factor 2
OXGR1: Oxoglutarate receptor 1
*P. aeruginosa*: *Pseudomonas aeruginosa*
PAMPs: Pathogen-associated molecular patterns
PRDX5: Peroxiredoxin 5
PrpB: Methylisocitrate lyase
PurF: Amidophosphoribosyltransferase F
*ripA*: Itaconate CoA-transferase RipA/Ict
*ripB*: (R)-specific enoyl-CoA hydratase RipB/Ich
*ripC*: Itaconate degradation C-C-lyase RipC
RipR: Itaconate degradation transcriptional regulator RipR
ROS: Reactive oxygen species
*S. aureus*: *Staphylococcus aureus*
SCS: Succinyl-CoA synthetase
SCV: *Salmonella*-containing vacuole
SDH: Succinate dehydrogenase
SIgA: Secretory immunoglobulin A
SigB: RNA polymerase sigma factor SigB
SLC13A3: Solute carrier family 13 member 3
STAT: Signal transducer and activator of transcription
STING: Stimulator of interferon genes
*S. *Typhimurium: *Salmonella enterica* serovar Typhimurium
TBK1: TANK binding kinase 1
TCA: Tricarboxylic acid
TET2: Tet methylcytosine dioxygenase 2
TFEB: Transcription factor EB
TLR: Toll-like receptor
UBR5: Ubiquitin protein ligase E3 component N-recognin 5
*V. cholerae*: *Vibrio cholerae*
*Y. pestis*: *Yersinia pestis*
